# Distal regulatory elements identified by methylation and hydroxymethylation haplotype blocks from mouse brain

**DOI:** 10.1186/s13072-018-0248-3

**Published:** 2018-12-29

**Authors:** Qin Ma, Zhengzheng Xu, Huan Lu, Ziying Xu, Yuanyuan Zhou, Bifeng Yuan, Weimin Ci

**Affiliations:** 1 Key Laboratory of Genomics and Precision Medicine, Beijing Institute of Genomics, Chinese Academy of Sciences, Beijing, 100101 China; 2 Key Laboratory of Analytical Chemistry for Biology and Medicine (Ministry of Education), Department of Chemistry, Wuhan University, Wuhan, 430072 China; 3 University of Chinese Academy of Sciences, Beijing, 100049 China; 4 Institute of Stem Cell and Regeneration, Chinese Academy of Sciences, Beijing, 100101 China

**Keywords:** 5hmC, 5mC, Methylation haplotype block, Methylation haplotype load, Hydroxymethylation haplotype block

## Abstract

**Background:**

5-Hydroxymethylcytosine (5hmC) is an oxidation product of 5-methylcytosine (5mC), and adjacent CpG sites in mammalian genome can be co-methylated and co-hydroxymethylated due to the processivity of DNMT and TET enzymes.

**Results:**

We applied TAB-seq and oxBS-seq to selectively detect 5hmC and 5mC at base resolution in the mouse cortex, olfactory bulb and cerebellum tissues. We found that majority of the called 5hmC CpG sites frequently have 5mC modification simultaneously and are enriched in gene body regions of neuron development-related genes in brain tissues. Strikingly, by a systematic search of regions that show highly coordinated methylation and hydroxymethylation (MHBs and hMHBs), we found that MHBs significantly overlapped with hMHBs in gene body regions. Moreover, using a metric called methylation haplotype load, we defined a subset of 1361 tissue-specific MHBs and 3818 shared MHBs. Shared MHBs with low MHL correspond with developmental enhancers, and tissue-specific MHBs resemble the regulatory elements for tissue identity.

**Conclusions:**

Our results provide new insights into the role of coordinately oxidized 5mC to 5hmC as distal regulatory elements may involve in regulating tissue identity.

**Electronic supplementary material:**

The online version of this article (10.1186/s13072-018-0248-3) contains supplementary material, which is available to authorized users.

## Background

The oxidation of 5mC to 5hmC is carried out by the 10–11 translocation (TET) enzymes [1]. A number of pathways to the removal of the methyl group of 5mC via 5hmC have been suggested and validated [2, 3], and thus, 5hmC is proposed as an intermediate of DNA demethylation. However, by mapping base-resolution methylomes in 17 adult mouse tissues with BS-seq method, previous study identified some tissue-specific hypomethylated regions which mark enhancers that are active in embryonic development but dormant in adult tissues [4]. Additionally, the oxidation of 5mC to 5hmC also occurs in brain at regulatory elements [5]. These results raise further questions that oxidation 5mC to 5hmC but not DNA hypomethylation may be required for tissue identity.

But 5hmC is cell-type specific and the pattern can be harnessed for analyzing heterogeneous samples. The small differences at single-CpG site between tissue types which could be important markers for small subpopulations in these tissues will be within the error range of the TAB-seq (TET-assisted bisulfite sequencing) method [6]. Strikingly, recent study has demonstrated superior sensitivity in detecting tissue-specific pattern with consideration of coordinated methylation of neighboring CpGs [7]. Due to the locally coordinated activities of DNMT and TET dioxygenase proteins, adjacent CpG sites on the same DNA molecule can share similar methylation and hydroxymethylation statuses. Thus, the analysis of CpG co-methylation and co-hydroxymethylation in cell populations within a tissue may aid in the deconvolution of heterogeneous tissue samples and elucidate the key regulatory elements for tissue identity. Therefore, the theoretical framework of linkage disequilibrium [8], which was developed to model the co-segregation of adjacent genetic variants on chromosomes in human populations, can be applied to the analysis of CpG co-methylation and co-hydroxymethylation in the cell populations of a tissue. However, single-base resolution profiling of both 5mC and 5hmC is crucial to understanding the functional role of the turnover from 5mC to 5hmC.

To the best of our knowledge, TAB-seq or oxBS-seq (oxidative bisulfite sequencing) [9] are the only currently available methods that can selectively detect 5hmC and 5mC, respectively, at base resolution. Herein, we applied the oxBS-seq and TAB-seq to profile 5mC and 5hmC at the single-nucleotide level in three brain tissues, including the cortex, olfactory bulb and cerebellum. Our results supported that a prominent role of coordinately oxidized 5mC to 5hmC as distal regulatory elements may involve in regulating tissue identity.

## Results

### Oxidization of 5mC to 5hmC but not global cytosine modification level characterizes brain tissues

**Fig. 1:**
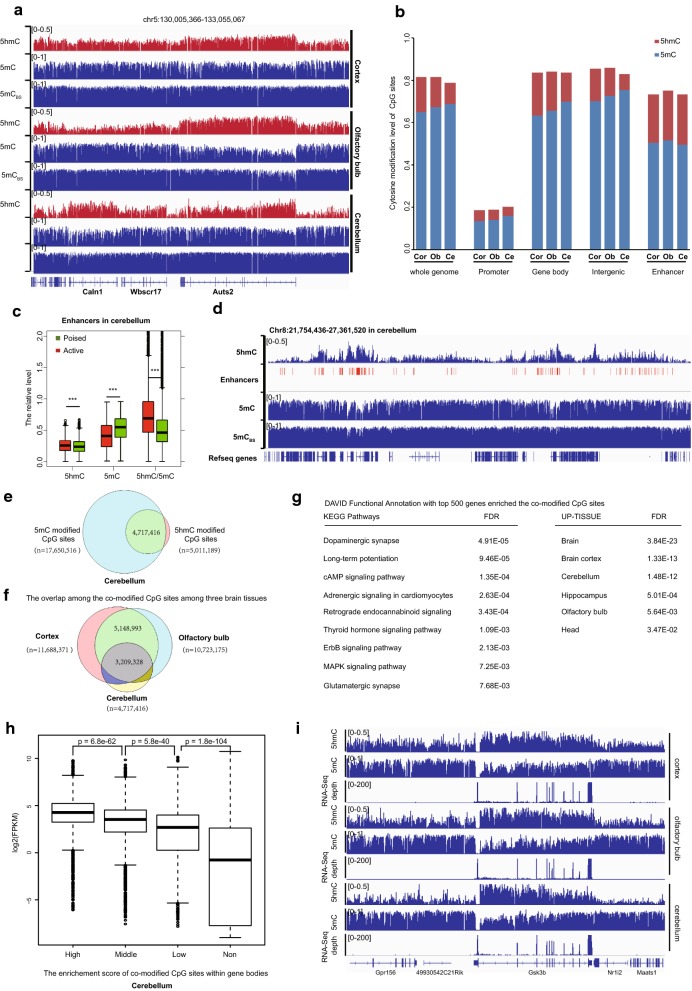
Oxidization of 5mC to 5hmC occurs globally at gene bodies for brain development-related genes. **a** Wiggle tracks of the 5hmC, 5mC and total modification (5hmC + 5mC) profiles in a genomic region on chromosome 5. **b** Average 5hmC and 5mC levels in different genomic elements determined by TAB-seq and oxBS-seq, respectively. The promoter is defined as being 500 bp upstream of the TSS. **c** Box plots of the 5hmC, 5mC and 5hmC/5mC levels in the active enhancers and poised enhancers in the cerebellum. **d** Wiggle tracks of the 5hmC, 5mC and total modification (5hmC + 5mC) profiles for a representative region with enriched enhancers. **e** Venn plot of the co-occurrence of 5hmC and 5mC at the same CpG site in the cerebellum. **f** Venn plot of the overlap for the co-modified CpG sites among the three brain tissues. **g** The DAVID Functional Annotation for the top 500 genes enriched in the co-modified CpG sites among the three brain tissues. **h** The association between gene expression level and the enrichment score of the co-modified CpG sites within gene body regions. The enrichment score was calculated as described in “Methods” section. Non represents that the enrichment score is less than 0. Statistical significances were evaluated by rank-sum test. **i** Graphical representation of the association between gene expression (depths of RNA-seq) and the average 5hmC, 5mC level in *gsk3b* gene locus

Firstly, we performed TAB-seq and oxBS-seq on cerebellum, cortex and olfactory bulb tissues derived from female mice. We sequenced to an average depth of 23-fold genomic coverage per tissue (Additional file 2: Table S1). Biological replicates were included for each tissue sample from an independent mouse. The principal component analysis (PCA) plot clearly showed that both hydroxymethylomes and methylomes stored memory of the tissue identities with great concordance among replicates (Additional file 1: Figure S1A). Consistent with previous study, we observed non-CpG methylation in all three brain tissues but the majority of 5hmC and 5mC exist in a CpG context (Additional file 1: Figure S1B and S1C). Next, we focused on the 5hmC and 5mC modifications in the CpG context. As exampled in the *Auts2* gene locus, although the overall modification (5mC + 5hmC) was similar among the three brain tissues as determined by the BS-seq method [4], the extent and/or distribution of oxidized 5mC to 5hmC were different among tissues (Fig. 1a). The relative 5mC and 5hmC levels in the three tissues are consistent with the results from mass spectrometry (Additional file 1: Figure S1D).

Moreover, both 5mC and 5hmC modifications are identified in all genomic elements, especially enhancers (Fig. 1b). We found that in contrast to the relative depletion of 5mC, 5hmC CpG sites were enriched in the enhancer regions (Fig. 1b and Additional file 1: Figure S1E). Thus, oxidization of 5mC to 5hmC but not DNA demethylation (hypomethylation) may also be required for enhancer activity which will escape from the detection of BS-seq method [10, 11]. To address this, we compiled the available enhancer data for the three brain tissues from the mouse Encyclopedia of DNA Elements (ENCODE) Project [12]. As predicted, although the global hypomethylation can significantly distinguish the active enhancers from poised enhancers, active enhancers also showed significantly higher levels of 5hmC/5mC compared to poised enhancers (Fig. 1c). A representative locus is shown in Fig. 1d. Thus, oxidized 5mC to 5hmC may play an important role in tissue identity, at least partially through the regulation of enhancer activity.

More importantly, with the single-nucleotide resolution mappings of both 5mC and 5hmC, we found that majority of the called 5hmC CpG sites frequently have 5mC modification simultaneously (co-modified sites) in all three brain tissues (Fig. 1e). Given that 5mC and 5hmC are binary for any particular cytosine, the co-occurrence of the two modifications indicates that the oxidization of 5mC to 5hmC occurs in a subset of cells within the tissues. Consistent with this scenario, we found that the co-modified CpG sites were not randomly distributed in brain tissues and showed great concordance among three brain tissues (Fig. 1f). Furthermore, DAVID functional annotation for the genes that enriched the shared co-modified sites among the three tissues revealed multiple neuron development-related KEGG pathways, such as dopaminergic synapse, retrograde endocannabinoid signaling and glutamatergic synapse (Fig. 1g). And these genes were also specifically expressed in the brain (Fig. 1g, DAVID Functional Annotation, UP-TISSUE). In contrast, the most enriched KEGG pathway is drug metabolism for the top 500 genes that enriched the 5mC-modified CpG sites in three brain tissues. And these genes were specifically expressed in the unfertilized egg (Additional file 1: Figure S1F and S1G). More importantly, further analyses including RNA-seq data (Additional file 2: Table S2) showed that the genes that enriched co-modified CpG sites within gene body regions were expressed higher compared to the genes without enrichments (Fig. 1h and Additional file 1: Figure S1H). A representative locus at *GSK3B* gene, a serine–threonine kinase involved in neuronal cell development [13], is shown in Fig. 1i. Collectively, oxidization of 5mC to 5hmC but not global modification level may regulate the brain tissue identity.

### Identification of methylation and hydroxymethylation haplotype blocks that significantly overlapped and co-localized with the regulatory elements

**Fig. 2:**
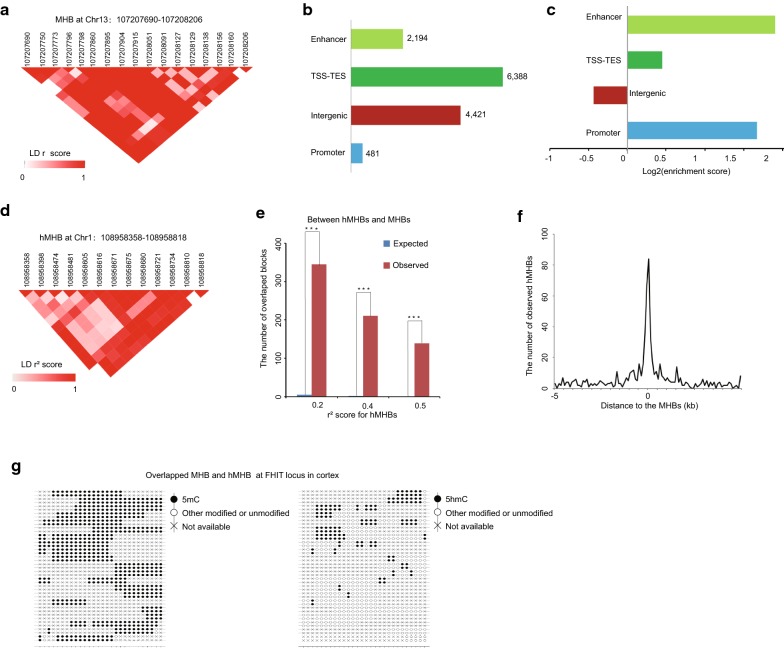
Characterization of the methylation haplotype blocks (MHBs) and tissue clustering based on methylation haplotype load. **a** An example of a MHB at the gene body of the gene at chromosome 13. The LD *r*^2^ scores between the CpG sites in the MHB were represented in heatmap. The scale bar from white to red represents the LD *r*^2^ scores between the two CpG sites from low to high. **b** Co-localization of MHBs (*n* = 10,809) with known genomic elements. **c** Enrichment of MHBs in known genomic features. **d** An example of a hMHB at Chromosome 1. The LD *r*^2^ scores between the CpG sites in the MHB were represented in heatmap. The scale bar from white to red represents the LD *r*^2^ scores between the two CpG sites from low to high. **e** The overlap between the hydroxymethylation haplotype blocks (hMHBs) and MHBs in gene body regions. The expected number of overlapped blocks was evaluated by random sampling method. The coordination was measured under different cutoffs for the LD *r*^2^ score of linkage disequilibrium two adjacent CpG sites hydroxymethylation. *P* values were calculated by hypergeometric test. ***Represents *p* value < 0.0001. **f** The observed number of the segment middle for hMHBs within 5 k of the segment middle for MHBs within gene body regions. The number for the segment middle for hMHBs was evaluated in the 100 bp bin. **g** Lollipop plots showing the 5mC and 5hmC modification of neighboring CpGs of a DNA molecule at FHIT locus, overlapped MHB and hMHB

5mC is cell-type specific, and the pattern of small subpopulation cells which may be important markers for the tissue type will hardly be identified due to the technical noise in measuring single-CpG methylation. Recently, Guo et al. [7] have demonstrated superior sensitivity with consideration of coordinated methylation of neighboring CpGs in detecting tissue-specific pattern. However, most of the recent efforts rely on the measurement of 5mC level by BS-seq method which cannot capture the oxidation of 5hmC to 5hmC without changing the overall cytosine level. Thus, an unbiased methylation status of multiple CpG sites in paired Illumina sequencing reads of oxBS-seq was extracted to form methylation haplotypes, and a pairwise “linkage disequilibrium” of CpG methylation LD *r*^2^ was calculated as previously reported [7]. We partitioned the genome into blocks of tightly coupled CpG methylation sites (which we refer to as MHBs; a representative MHB is shown in Fig. 2a) using LD *r*^2^ cutoff of 0.5. We identified 10,809 MHBs with an average size of 501 bp and a minimum of four CpGs per block, which tended to be tightly co-regulated on the epigenetic status at the level of single DNA molecules (Additional file 1: Figure S2A and S2B; Additional file 3: Table S3). The majority of the CpG sites within the same MHBs were nearly perfectly coupled (LD *r*^2^ ~ 1.0). The MHBs established by the oxBS-seq data represent a distinct type of genomic feature that partially overlaps with known genomic elements. Among all of the MHBs, 4421 (40.9%) were located in intergenic regions, whereas 6388 (59.1%) regions were located in transcribed regions (Fig. 2b). These MHBs were significantly enriched in promoters, gene body regions and enhancers (Fig. 2c). Collectively, coordinately 5mC at block level that are directly or indirectly coupled to transcriptional regulation.

To further prove the oxidization of 5mC to 5hmC also occurring in coordinated manner, we examined whether the MHBs were overlapped with hydroxymethylation haplotype blocks (hMHBs) as defined by the TAB-seq data (see “Methods” section). We focused on gene body regions since the 5hmC level is extremely low in intergenic regions within the error rate of TAB-seq method. We partitioned the genome into blocks of tightly coupled CpG hydroxymethylation sites using LD *r*^2^ cutoff of 0.2. We identified 2296 hMHBs which tended to be tightly co-regulated on the epigenetic status at the level of single DNA molecules (Additional file 5: Table S5). A representative hMHB at chromosome 1 is shown in Fig. 2d. As expected, we did find that the hMHBs were significantly overlapped with MHBs in gene body regions (Fig. 2e). To more precisely quantify resolution, we calculated the cumulative distribution of distance between MHB and hMHBs in gene body regions. And majority of hMHBs were co-localized with MHBs for distances less than 500 bp (Fig. 2f). As shown in Fig. 2g, within the same locus, both tightly coupled methylated CpGs and hydroxymethylated CpGs statuses were identified at the level of individual reads. This indicated coordinated methylation and hydroxymethylation of neighboring CpGs allowed for the detection of small subpopulations differences within tissue.

### Block-level analysis using methylation haplotype load can aid in the deconvolution of heterogeneous brain tissues

**Fig. 3:**
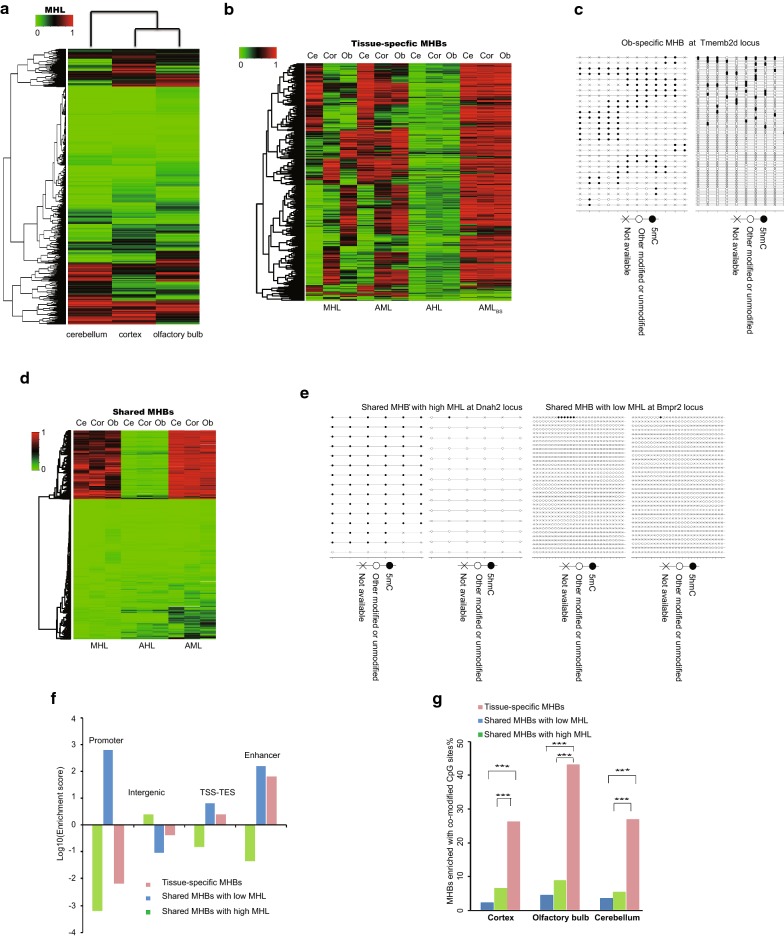
Tissue-specific co-methylation regions capture the local co-hydroxymethylation regions. **a** MHL-based unsupervised clustering of mouse cerebellum, cortex and olfactory bulb using all MHBs. **b** Comparison of the cluster performance to the brain tissues using the MHL, the average methylation level (AML), the average hydroxymethylation level (AHL) and the average methylation level from BS-seq (AML_BS_) metrics in the tissue-specific MHBs. **c** Lollipop plots showing the 5mC and 5hmC modification of neighboring CpGs of a DNA molecule within a representative olfactory bulb-specific MHB. **d** Shared MHBs showed bimodal distribution of MHL and low level of 5hmC. AHL: average hydroxymethylation level, AML: average methylation level. **e** Lollipop plots showing the 5mC and 5hmC modification of neighboring CpGs of a DNA molecule (or a sequencing read) within two representative shared MHBs in olfactory bulb. **f** Enrichment of tissue-specific MHBs and shared MHBs with low or high MHLs in known genomic features. **g** The percentages of MHBs with enriched co-modified CpG sites for tissue-specific MHBs and shared MHBs with low or high MHLs in the three tissues. Statistical significance is evaluated by hypergeometric test. ***Represents *p* value < 0.0001

To enable a quantitative analysis of the co-methylation patterns within individual MHBs across samples, we used the same metric called methylation haplotype load (MHL) as the weighted mean of the fraction of fully methylated haplotypes and substrings at different lengths [7]. Consistent with a previous study [7], the block-level analysis based on the MHL distinguished these three brain tissues (Fig. 3a). To identify a subset of MHBs for effective clustering of these three brain tissues, we calculated a tissue-specific index (TSI) for each MHB (“Methods” section). We identified a set of 3818 shared MHBs and 1361 tissue-specific MHBs (Additional file 4: Table S4). Within these tissue-specific MHBs, we compared the performance for effective clustering of the different tissues between the average methylation level (AML), the average hydroxymethylation level (AHL) and the average methylation level (AML_BS_) from published BS-seq data (Fig. 3b) [4]. A subset of tissue-specific MHBs can also be identified by AML (Fig. 3b). However, the clustering performance of AHL was limited by the technical noise and sensitively in measuring single-CpG hydroxymethylation. And AML_BS_ in the blocks had the worst performance for clustering tissue-specific MHBs (Fig. 3b). Collectively, oxidization of 5mC to 5hmC but not global modification level may be the key feature for tissue-specific MHBs which will escape from the detection of BS-seq method.

### Tissue-specific MHBs but not shared MHBs capture the local co-modified regions

To evaluate whether the small subpopulations differences could be important markers for tissue type, we examined whether the tissue-specific MHBs but not shared MHBs capture the local co-modified CpGs. Strikingly, we found that the tissue-specific MHBs (*n* = 1361) have a high MHL, the highest level of 5hmC and moderate levels of 5mC, which were specifically enriched in the enhancer regions (Additional file 1: Figure S2C and Fig. 3f). This is consistent with scenario that oxidization of 5mC to 5hmC may act as new regulatory elements in regulating tissue identity. A representative olfactory bulb-specific MHB is shown in Fig. 3c. In contrast, the shared MHB showed a bimodal distribution of the MHL (1257 for MHB with high MHL, 2561 for MHB with low MHL) and low level of 5hmC (Fig. 3d). The shared MHBs with high MHLs had the highest 5mC level and a residual 5hmC level, which were enriched in the intergenic regions (Additional file 1: Figure S2C and Fig. 3f). The shared MHBs with low MHLs had both the lowest 5mC and 5hmC levels, which were enriched in promoter regions (Additional file 1: Figure S2C and Fig. 3f). Two representative shared MHBs are shown in Fig. 3e. Furthermore, we identified a significant enrichment of co-modified CpG sites in the tissue-specific MHBs but not in the shared MHBs in all three tissues (Fig. 3g). Collectively, tissue-specific MHBs but not shared MHBs capture the local co-modified regions.

### Shared MHBs with low MHL, but not tissue-specific MHBs, correspond with developmental enhancers

**Fig. 4:**
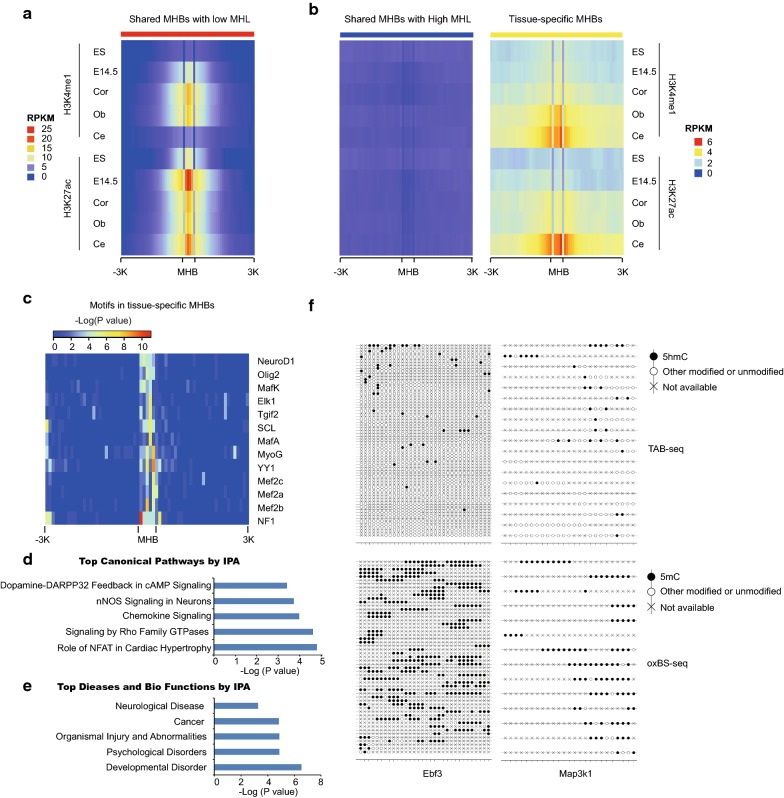
Tissue-specific MHBs resembled the regulatory elements for tissue identity. **a** Heatmap representing the enrichment of H3K4me1 and H3k27ac relative to the shared MHBs with low MHL in adult tissues, mouse embryonic stem cells and the whole brain tissue from E14.5 embryo. **b** The enrichment of H3K4me1 and H3k27ac relative to the shared MHBs with high MHL and tissue-specific MHBs. **c** Transcriptional factor binding motif enrichments near tissue-specific MHBs. **d**, **e** The IPA analyses of the genes overlapped with tissue-specific MHBs found canonical pathways and diseases significantly affected. **f** Lollipop plots showing the 5mC and 5hmC modification of co-hyroxymethylation of neighboring CpGs of a DNA molecule (or a sequencing read) within cerebellum-specific MHBs

Given MHBs are enriched in enhancer regions, we asked whether MHBs are regulatory elements which may regulate brain development. Then, we compared them to publicly available genomic annotations. We found that shared MHBs with low MHL were enriched in active enhancers in embryonic stem cells and developing brain from E14.5 (Data from ENCODE), and shared MHBs with high MHL were not enriched in active enhancers (Fig. 4a, b). These results are consistent with recent findings that some hypomethylated regulatory elements are dormant in adult tissue but active in embryonic development [4]. In contrast, tissue-specific MHBs were enriched for stronger enhancers in adult tissues compared to embryonic stem cells and the whole brain from embryonic day E14.5 (Fig. 4a, b). Collectively, shared MHBs with low MHL correspond with developmental enhancers, but tissue-specific MHBs were enriched in active enhancers in adult tissues.

### Tissue-specific MHBs resemble the regulatory elements for tissue identity

To further prove whether tissue-specific MHBs are regulatory sequences, we examined them for the enrichment of transcription factor binding motifs. Indeed, consensus motifs for known brain-specific master regulators were significantly enriched in the tissue-specific MHBs. For example, motifs for neuronal development factors NeuroD1, Olig2 and MEF2A, B, and D were specifically identified (Fig. 4c). All of these factors are involved in synaptic differentiation and neural crest differentiation [14, 15]. Next, we used the Ingenuity Pathway Analysis (IPA) to predict functions of the genes overlapped with tissue-specific MHBs. As expected, multiple neuron development-related pathways were identified, such as Dopamine-DARPP32 Feedback in cAMP Signaling and nNOS Signaling in Neurons (Fig. 4d). And further top diseases and biofunctions also supported these genes may involve in neurological disease and cancer (Fig. 4e).

We further evaluated whether tissue-specific MHB blocks will detect the small differences in subpopulations within tissue. We integrated the data of single-cell RNA profiling of the mouse cerebellum [16]. Among the genes overlapped with cerebellum-specific MHBs, Ebf3 and Map3k1 have been shown as top ten highest expressed genes in cerebellum granule precursor cells. As shown in Fig. 4f, a small percentage of reads showed co-hyroxymethylation of neighboring CpGs within cerebellum-specific MHBs of the two important genes for small subpopulations in cerebellum. Collectively, tissue-specific MHBs may correspond to the regulatory elements for tissue identity.

## Discussion

A recent study showed that the identification of methylation haplotype blocks aids in the deconvolution of heterogeneous tissue [7]. Herein, we extended the established analysis of co-methylated CpG patterns with the oxBS-seq data. Although the mathematical representations are identical, one major difference is that the oxBS-seq method can unbiasedly measure the 5mC level instead of the sum of 5mC and 5hmC for the BS-seq data. Additionally, we also extended the analysis to TAB-seq data; one major difference is that co-hydroxymethylation analysis must focus on gene body regions since the 5hmC level is extremely low beyond gene body regions and within the error range of the TAB-seq method. These regions will be false positively called as co-hydroxymethylation regions with low modification level if included. Future studies will be required to precisely define the cell-type-specific co-methylation/co-hydroxymethylation patterns in the heterogeneous brain tissues.

Herein, we found that oxidization of 5mC to 5hmC at block level as potential regulatory elements can better separate three closely related brain tissues. There are two possible explanations: (1) BS-seq cannot capture the oxidation of 5hmC to 5hmC without changing the overall cytosine modification level and (2) consideration of coordinated methylation and hydroxymethylation of neighboring CpGs allowed for the detection of small differences between tissue types which could be important markers for small subpopulations in these tissues.

Furthermore, recent studies [17] propose that epigenetic changes could result from prior events dictated by genetic information, such as TF binding. The methylated DNA binding factors, the potential pioneer factors, may bind chromatin (highly methylated) and turn it into a regulatory element through the turnover to 5hmC [18]. Consistently, a recent study showed that many members of TFs, such as NeuroD1 and the extended homeodomain family, preferred to bind to mCpG-containing sequences. And TFs that preferred mCpG are commonly involved in embryonic and organismal developmental processes [19]. Strikingly, in our study, NeuroD1 and homeodomain TF, TGIF2, are also identified in the tissue-specific MHBs. Thus, these transcription factors may act as the pioneer factors in establishing the regulatory elements for tissue identity. However, at this point, we do not show that this phenomenon can go beyond correlation. The genetic deletion of the potential pioneer factors would be needed to further support this hypothesis. Investigation is also warranted to determine whether the presence of hydroxymethylation is actually involved in the activity of these potential regulatory elements through specific readers. Additionally, pronounced reciprocal 5mC and 5hmC changes are also identified at cancer-related genes during tumorigenesis [20]. Thus, the theoretical framework can be applied to identify the biomarkers for cancer-specific pathologies that may be valuable in further understanding cancer biology, diagnosis and therapy. Moreover, it will be vital in future studies to use methods that distinguish 5mC from 5hmC, such as oxBS-seq, when profiling DNA methylation in the genome.

## Conclusion

We presented the first dataset that allows for a clear separation of 5mC and 5hmC at the single-molecule level (or a sequencing read) across the whole genome. We showed that oxidation of 5mC to 5hmC can occur in a coordinated manner in mouse brain tissues and identified haplotype blocks for both 5mC and 5hmC. Furthermore, through examining the co-localization with markers of enhancers in mouse embryonic and adult tissues, the enrichment of TF motifs and through pathway analysis, we revealed an interesting phenomenon that shared MHBs with low methylation (MHL) tend to overlap with enhancers active during brain development, whereas tissue-specific MHBs (among the three brain regions) tend to co-localize with enhancers active in adult brain. Collectively, our results provide new insights into the role of coordinately oxidized 5mC to 5hmC as distal regulatory elements may involve in regulating tissue identity.

## Methods

### Tissue samples and genomic DNA extraction

The cerebellum, cortex and olfactory bulb were dissected from an 8-week-old female C57Bl/6 mouse. The cortex tissue came from the whole cerebral cortex excluding the hippocampus regions. The cerebellum and olfactory bulb tissues came from the whole tissues, respectively. The tissues for the technical replicates were from independent mice, and at least two replicates were included in the different assays as indicated. Genomic DNA was isolated using a DNA extraction kit (Qiagen, Cat#: 51306) according to the manufacturers’ instructions.

### LC–ESI–MS analysis

The LC–ESI–MS analysis was performed according to our previous study [20]. Briefly, the percentages of 5mC and 5hmC were calculated by the following formula, where M (cytosine), M (5mC) and M (5hmC) are the molar quantities of cytosine.

$$5{\text{mC \% }} = \frac{{{\text{M}}\left( {5{\text{mC}}} \right)}}{{{\text{M}}\left( {\text{cytosine}} \right) + {\text{M}}\left( {5{\text{mC}}} \right) + {\text{M}}\left( {5{\text{hmC}}} \right)}} \times 100$$

$$5{\text{hmC \% }} = \frac{{{\text{M}}\left( {5{\text{hmC}}} \right)}}{{{\text{M}}\left( {\text{cytosine}} \right) + {\text{M}}\left( {5{\text{mC}}} \right) + {\text{M}}\left( {5{\text{hmC}}} \right)}} \times 100$$

### oxBS-seq

The sequencing libraries were constructed as described [9] with minor modifications. Briefly, genomic DNA (0.5–1 μg) was end-repaired, A-tailed and ligated to methylated adaptors following the manufacturer’s instructions. The ligated fragments were then purified using the Bio-Rad Micro Bio-Spin P-6 SSC column (SSC buffer). After the purification, the samples were denatured using 1 M NaOH and oxidized using an oxidant solution (15 mM KRuO_4_ in 0.05 M NaOH). After the purification, the DNA underwent bisulfite conversion using the EZ DNA Methylation Gold kit (Zymo Research) according to the instruction manual. The library was sequenced using Illumina HiSeq X Ten. The paired reads were uniquely mapped to the reference genome (mm10, UCSC) by Bismark. The efficient conversion of 5hmC to uracil was calculated by a spiked 5hmC control from Zymo Research (Cat#: D5405).

### TAB-seq

The sequencing libraries were constructed as described [6] with minor modifications. Briefly, genomic DNA, with spike-in controls, was glycosylated and oxidized using the kit from Wisegene (Cat#: K001). Then, the DNA underwent bisulfite conversion using the EZ DNA Methylation Gold kit (Zymo Research) according to the instruction manual. The library was sequenced using Illumina HiSeq X Ten. The paired reads were mapped uniquely to the reference genome (mm10, UCSC) by Bismark. The efficient conversion of unmodified cytosine to uracil and the efficient conversion of 5mC to 5caU/U were calculated using spiked M.SssI-treated lambda DNA.

### The associations between the biological replicates for TAB-seq and oxBS-seq

The 5hmC and 5mC levels of the biological replicates were calculated in 1 Mb regions throughout the genome. Then, a hierarchical cluster analysis was performed using hcluster in R with ward.D methods.

### Quantification of the 5hmC and 5mC levels of each CpG site

The 5hmC level or the 5mC level of each CpG site was calculated using the same methods as described [20]. Briefly, a binomial distribution model was performed to calculate the significance for the hydroxymethylation or methylation. Only sites with Benjamini–Hochberg-corrected binomial *P* value ≤ 0.05 and reads coverage ≥ 5 were considered hydroxymethylated or methylated. We counted the number of “C” bases from the sequencing reads as hydroxymethylated or methylated (denoted as *N*_C_) and the number of “T” bases as unmodified (denoted as *N*_T_). The hydroxymethylation level or methylation level was estimated as *N*_C_/(*N*_C_ + *N*_T_).

### The enrichment scores of the 5hmC- and 5mC-modified sites in the different genomic regions

The enrichment score was calculated by the following formula: the enrichment score_in the genomic element_ = log2 (# called modified sites_in the genomic element_/# expected). # expected was computed as: # called modified sites_in the genome_ × # CpG sites_in the genomic element_/# total CpG sites_in the genome_. # denotes the number of sites.

### Identification of the genes that enriched the co-modified CpG sites

First, the CpG sites that were simultaneously identified as 5hmC- and 5mC-modified sites were determined as the co-modified CpG sites. Then, the enrichment score of the sites in each gene was calculated by the following formula:

The enrichment score_in the gene_ = log2 (#the co-modified CpG sites)_in the gene_/# expected). # expected was computed as: # the co-modified CpG sites_in the genome_ × # CpG sites_in the gene_/# total CpG sites_in the genome_. # denotes the number of sites.

### Identification of the methylation haplotype blocks (MHBs)

The MHBs were identified as described [7] with minor modifications. Briefly, the mouse genome was split into non-overlapping ‘sequenceable and mappable’ segments using the oxBS-seq data from the mouse cerebellum, cortex and olfactory bulb tissues. The “sequenceable and mappable” segments are defined as the high-quality mapping regions which were assembled from the pair-end reads. The mapped reads from the oxBS-seq datasets were converted into methylation haplotypes within each segment. The methylation linkage disequilibrium was calculated on the combined methylation haplotypes. We then partitioned each segment into MHBs. Candidate MHBs were defined as the genomic region in which the LD *r*^2^ value of two adjacent CpG sites was no less than 0.5. Then, the LD *r*^2^ matrix of the segments was calculated. The candidate MHBs were extended if the LD *r*^2^ value of the CpG sites in the segments was no less than 0.5.

### Identification of the hydroxymethylation haplotype blocks (hMHBs)

We identified hMHBs using the similar theoretical framework as MHBs with minor modifications. Briefly, for TAB-seq data, the readout of both methylated and unmodified sites will be “T,” while the readout of hydroxymethylated sites will be “C.” Thus, we focused on the gene body regions which enriched 5hmC modifications to identify the co-hydroxymethylation blocks (hMHBs). In this way, we can avoid the false positively called as co-hydroxymethylation blocks for the highly methylated regions (such as intergenic regions) and unmethylated regions (such as promoter regions and CpG islands) with TAB-seq data. Additionally, the average 5hmC level of each called CpG sites is lower than the average 5mC level; thus, we tried different cutoffs for the LD *r*^2^ score of linkage disequilibrium two adjacent CpG sites hydroxymethylation.

### The coordination between the hMHBs and MHBs in gene body regions

The coordination analysis was performed by random sampling method. First, the same amount of regions with same length distribution as MHBs within gene body regions were randomly sampled and repeated 10,000 times. Then, the overlap between randomly sampled regions and the hMHBs was calculated as the expected number of overlapped blocks between hMHBs and MHBs within gene body regions. Statistical significance was calculated by hypergeometric test.

### Enrichment analysis of the methylation haplotype blocks for known genomic regulatory elements

The enrichment analysis was performed by random sampling as previously described [21]. Genomic regions with the same number of MHBs and CpGs were randomly sampled within the mappable CpG regions and repeated 10,000 times. Then, we obtained the expected MHBs in the genomic element. All of the genomic coordinates were based on the mm10 mouse genomic sequence.

The enrichment score was calculated by the following formula:

The enrichment score_in the genomic element_ = log2 (# called MHBs_in the genomic element_/# expected).

### Methylation haplotype load (MHL)

The MHLs were identified as described [7], which is the normalized fraction of methylated haplotypes at different lengths.

### Identification of tissue-specific MHBs

The tissue-specific MHBs were identified as described [7] with minor modifications. To investigate the tissue-specific MHBs, the tissue-specific index (TSI) was defined. An empirical threshold TSI > 0.6 was used to define the tissue-specific MHBs.

$${\text{TSI}} = \frac{{\mathop \sum \nolimits_{j = 1}^{n} 1 - \frac{{10^{{{\text{MHL(}}j )}} }}{{10^{{{\text{MHL}}\left( {\hbox{max} } \right)}} }}}}{n - 1}$$

where *n* indicates the number of the tissues, MHL(*j*) denotes the MHL of *j*th tissue and MHL max denotes the MHL of the highest methylated tissue.

### Enrichment analysis of the MHBs for the co-modified CpG sites

First, the co-modified CpG sites in each MHB were calculated. Then, the enrichment score of the co-modified CpG sites in each MHB was calculated by the following formula:

The enrichment score_in the MHB_ = log2 (#the co-modified CpG sites)_in the MHB_/# expected). # expected was computed as: # the co-modified CpG sites_in the genome_ × # CpG sites_in the MHB_/# total CpG sites_in the genome_. # denotes the number of sites.

### Motif analysis

We searched for the enrichment of known motifs using the Homer tool [22]. To search for the motifs within a single tissue, we used default parameters with a fragment size for motif searching of 200 bp.

### External datasets

According to the H3K4me1 and H3K27ac marks, two types of enhancers were distinguished as follows: active enhancers that were simultaneously marked by a distal H3K4me1 and H3K27ac and poised enhancers that were solely marked by a distal H3K4me1. The H3K4me1 and H3K27ac peaks of the adult mouse cortex, olfactory bulb and cerebellum tissues were acquired from the ENCODE project.

## Additional files

**Additional file 1.** Characteristics of MHBs in mouse brain tissues by base-resolution hydroxymethylome and methylome in the mouse brain tissue.

**Additional file 2.** Summary of single-base 5mC and 5hmC sequencing using oxBS-seq and TAB-seq.

**Additional file 3: Table S3.** Methylation haplotype block regions (mm10).

**Additional file 4: Table S4.** The classifications of MHBs.

**Additional file 5: Table S5.** Hydroxymethylation haplotype blocks (mm10).
