# Supplementary material for: Distal regulatory elements identified by methylation and hydroxymethylation haplotype blocks from mouse brain
Source: Epigenetics Chromatin. 2018 Dec 29;11:75. doi: 10.1186/s13072-018-0248-3 (PMC6311040; doi:10.1186/s13072-018-0248-3)
Supplement: Supplementary file 1 — Additional file 1. Characteristics of MHBs in mouse brain tissues by base-resolution hydroxymethylome and methylome in the mouse brain tissue. [file 13072_2018_248_MOESM1_ESM.pdf]

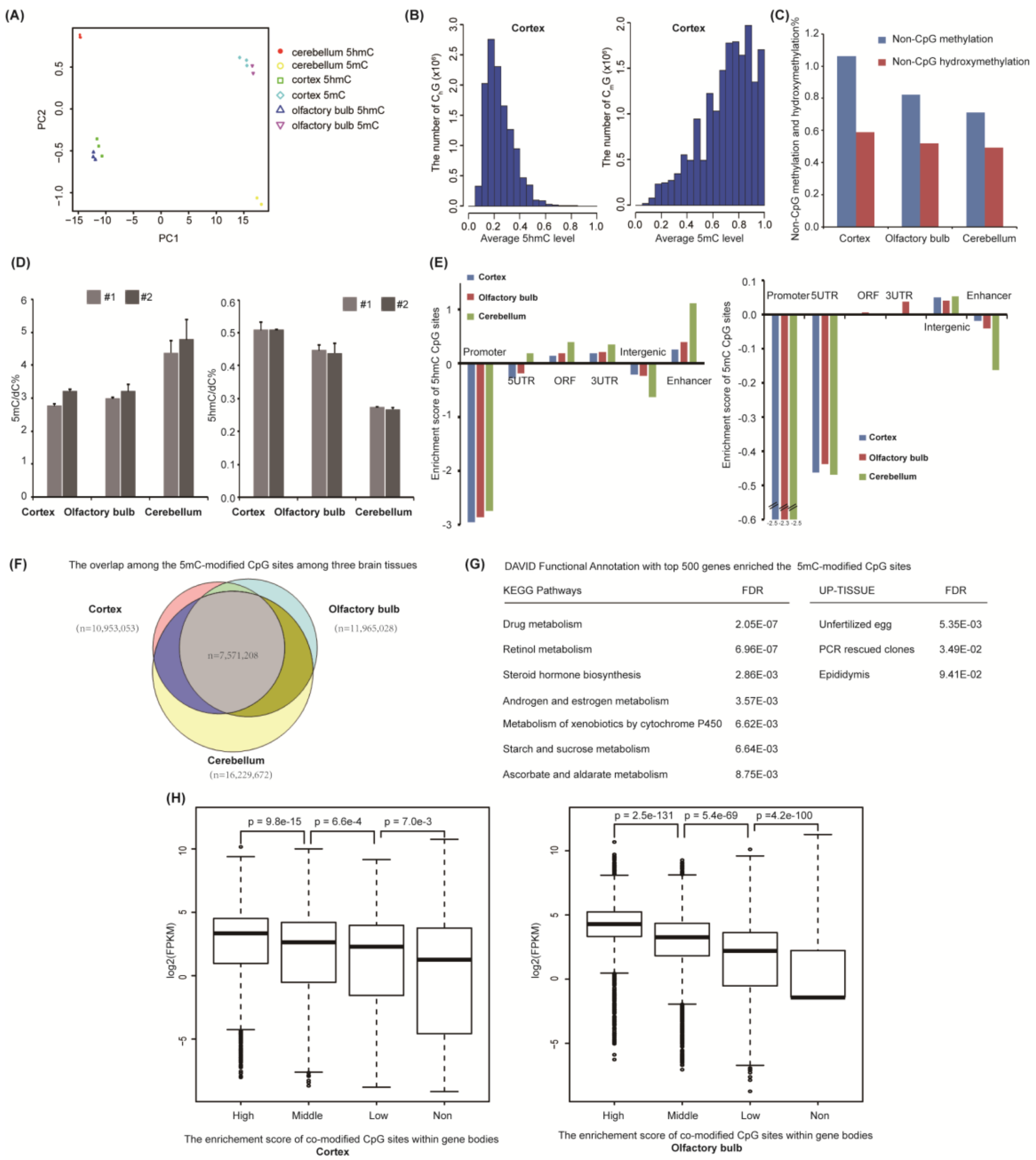

Figure S1. Base-resolution hydroxymethylome and methylome in the mouse brain tissues. A PCA plot of 5hmC and 5mC profiles among the biological replicates in brain tissues. 5hmC and 5mC levels calculated in the 1 Mb bin. **B** The distribution of estimated abundances of 5hmC at called 5hmC modified CpG sites and 5mC at called 5mC modified CpG sites in the cortex tissue determined by TAB-seq and oxBS-seq, respectively. **C** Average 5hmC and 5mC levels in the genome in non-CpG context determined by TAB-seq and oxBS-seq, respectively. **D** 5mC and 5hmC levels were evaluated by LC-ESI-MS. #1 and #2 represent two biological replicates. **E** The enrichment scores of called 5hmC and 5mC sites in different

genomic elements relative to expected in three tissues, respectively. Score > 0 was defined as enriched. **F** Venn plot of the overlap for the 5mC-modified CpG sites among the three brain tissues. **G** The DAVID Functional Annotation for the top 500 genes enriched in the 5mC-modified CpG sites among the three brain tissues. **H** The association between gene expression level and the enrichment score of co-modified CpG sites within gene body region. The enrichment score was calculated as described in Methods. Non represents that the enrichment score is less than 0. Statistical significances were evaluated by rank-sum test.

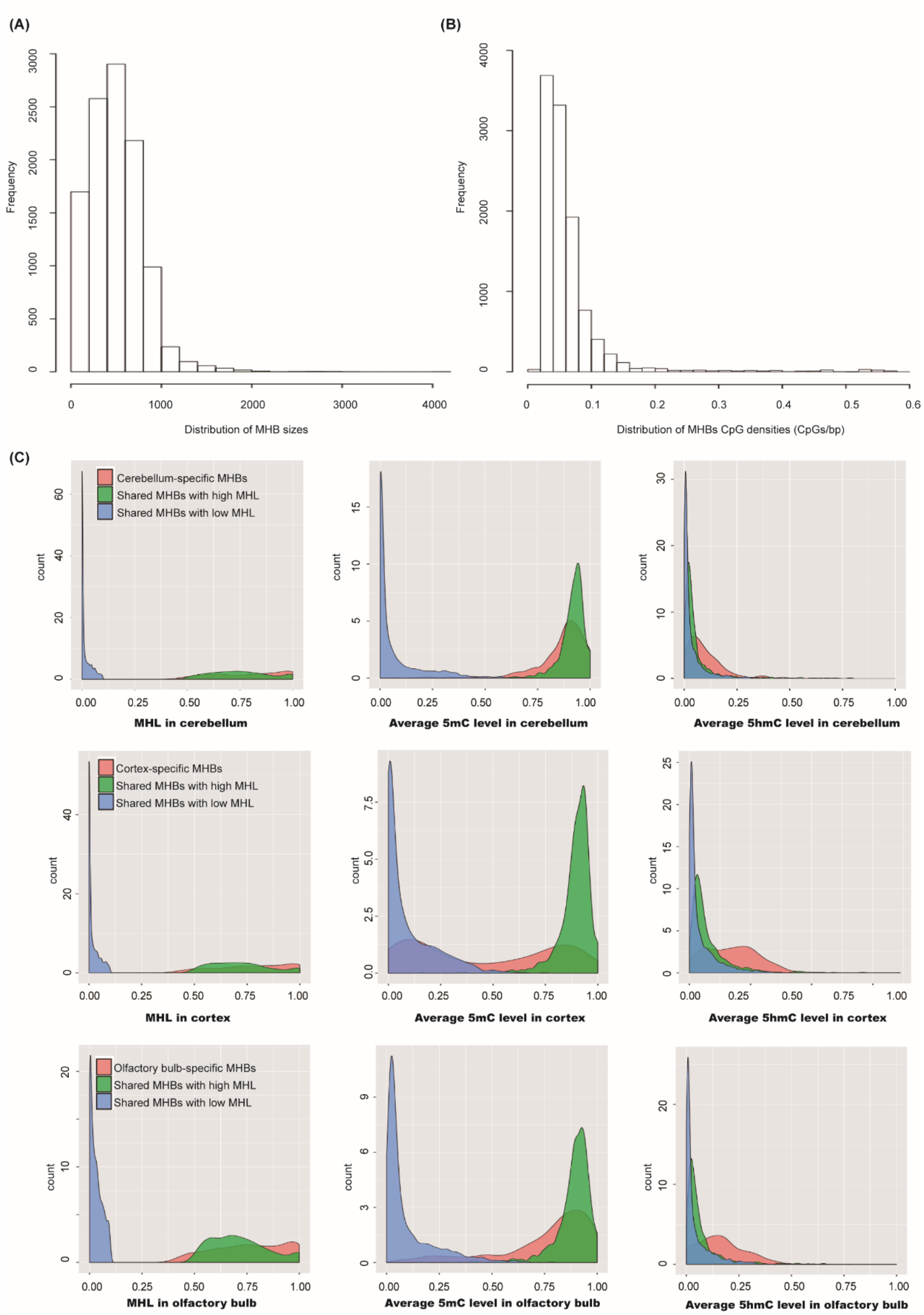

Figure S2. Characteristics of MHBs in mouse brain tissues. A Distribution of MHB sizes. **B** Distribution of MHBs CpG densities (CpGs/bp). **C** The density plots of the MHL, 5mC and 5hmC levels in tissue-specific MHBs and the shared MHBs with high MHLs or low MHLs in brain tissues.
