# Supplementary material for: Distal regulatory elements identified by methylation and hydroxymethylation haplotype blocks from mouse brain
Source: Epigenetics Chromatin. 2018 Dec 29;11:75. doi: 10.1186/s13072-018-0248-3 (PMC6311040; doi:10.1186/s13072-018-0248-3)
Supplement: Supplementary file 2 — Additional file 2. Summary of single-base 5mC and 5hmC sequencing using oxBS-seq and TAB-seq. [file 13072_2018_248_MOESM2_ESM.pdf]

Table S1 Summary of single-base 5mC and 5hmC sequencing using oxBS-seq and TAB-seq.

		Cortex	Olfactory bulb	Cerebellum
5hmC (TAB-Seq)	Bisulfite conversion rate	99.20%	99.53%	99.11%
	Tet oxidization rate	98.38%	98.45%	98.11%
	Protection rate	98. 93%	99.31%	99.14%
	Mapped depth	23	26	21
	#CpG covered > 5X	20.02M	20.23M	19.55M
	Average 5hmC level in CpG context	16.48%	14.60%	10.00%
5mC (oxBS-Seq)	Bisulfite conversion rate	99.85%	99.86%	99.85%
	Oxidization rate	99.00%	98.61%	98.95%
	Mapped depth	17	30	20
	#CpG covered > 5X	19.19M	20.16M	19.42M
	Average 5mC level in CpG context	64.93%	67.00%	68.78%

Both bisulfite conversion rate and oxidation rate by Tet was calculated by *M. SsI* treated spiked lambda DNA. Average 5hmC and 5mC level for all CpG sites is calculated as C/(C+T) for TAB-seq and oxBS-seq, respectively. M means million.

Table S2 Summary of RNA-seq.

	Total reads	Uniquely mapped reads	Uniquely mapped rate
Cortex replicate 1	12.15G	9.27G	76.30%
Cortex replicate 2	17.01 G	14.68G	86.30%
Olfactory bulb replicate 1	11.32G	9.11G	80.40%
Olfactory bulb replicate 2	12.53G	10.39G	82.90%
Cerebellum replicate 1	14.10G	12.25G	86.90%
Cerebellum replicate 2	8.60G	6.751	78.50%

The raw FASTQ data files were mapped to the mouse reference sequence (mm10) for mRNA analysis and gencode version 9 for ncRNA analysis using Tophat program. G means billion.
